# Supplementary material for: Active site specificity profiling datasets of matrix metalloproteinases (MMPs) 1, 2, 3, 7, 8, 9, 12, 13 and 14
Source: Data Brief. 2016 Feb 22;7:299–310. doi: 10.1016/j.dib.2016.02.036 (PMC4777984; doi:10.1016/j.dib.2016.02.036)
Supplement: Supplementary file 10 — Supplementary material [file mmc10.zip › WebPICS_hMMP1_T_1%/P2prime.html]

 

PICS results


|  |  |
| --- | --- |
| **P2prime\_A**  25 in 277 sites   9.0 %    effects > 10 perc. pnts.  (vice-versa in brackets)  P1\_A: 25.9 (13.0) |  |
| **P2prime\_H**  13 in 277 sites   4.7 %    effects > 10 perc. pnts.  (vice-versa in brackets)  P2\_W: 14.3 (62.0)   P1\_Q: 17.3 (14.1) |  |
| **P2prime\_I**  30 in 277 sites   10.8 %    effects > 10 perc. pnts.  (vice-versa in brackets)  P2\_Q: 14.4 (12.7) |  |
| **P2prime\_L**  44 in 277 sites   15.9 %    effects > 10 perc. pnts.  (vice-versa in brackets)  P1\_A: 11.4 (10.1)   P3prime\_D: 13.8 (19.6) |  |
| **P2prime\_M**  14 in 277 sites   5.1 %    effects > 10 perc. pnts.  (vice-versa in brackets)  P2\_M: 10.7 (14.9)   P1\_N: 24.5 (11.0) |  |
| **P2prime\_Q**  15 in 277 sites   5.4 %    effects > 10 perc. pnts.  (vice-versa in brackets)  P2\_A: 18.4 (12.0)   P3prime\_H: 16.8 (27.9) |  |
| **P2prime\_T**  18 in 277 sites   6.5 %    effects > 10 perc. pnts.  (vice-versa in brackets)  P2\_D: 19.1 (14.3)   P3prime\_S: 13.9 (10.9) |  |
| **P2prime\_V**  49 in 277 sites   17.7 %    effects > 10 perc. pnts.  (vice-versa in brackets)  P3prime\_T: 14.3 (41.1) |  |
